# 

**Published:** 1852-08

**Authors:** 


No. III. AUGUST. Vol. VIII.

### BUFFALO MEDICAL JOURNAL AND MONTHLY REVIEW OF MEDICAL AND SURGICAL SCIENCE.

EDITED BY AUSTIN FLINT, M. D.

BUFFALO:
PUBLISHED BY JEWETT, THOMAS & CO.
Commercial Advertiser Buildings.
1852

*Price* $2.50 *per annum . . . in Advance.*

